# Optimal Hospital Volume to Minimize Postoperative Mortality After Esophagectomy for Cancer in Low Population Density Countries: A Binational Study of Australia and New Zealand

**DOI:** 10.1002/wjs.12595

**Published:** 2025-04-17

**Authors:** Josipa Petric, Muktar Ahmed, Maziar Navidi, David Pilcher, Shailesh Bihari, Norma B. Bulamu, Tim Bright, David I. Watson

**Affiliations:** ^1^ Flinders Health and Medical Research Institute and College of Medicine and Public Health Flinders University Adelaide Australia; ^2^ Department of Surgery Flinders Medical Centre Adelaide Australia; ^3^ Department of Intensive Care Alfred Health Victoria Australia; ^4^ The ANZICS Centre for Outcome and Resources Evaluation Victoria Australia; ^5^ The Australian and New Zealand Intensive Care Research Centre School of Public Health and Preventive Medicine Monash University Victoria Australia; ^6^ Intensive and Critical Care Unit Flinders Medical Centre Adelaide Australia

**Keywords:** centralization, esophagectomy, length of stay, mortality, volume‐outcome

## Abstract

**Background:**

A relationship between hospital volume and postoperative mortality following esophagectomy for cancer has been reported in Europe and USA, leading to centralization of surgery for esophageal cancer in some countries. It is unclear if this is replicated in countries with low population density such as Australia and New Zealand (ANZ). This study determined the relationship between hospital volume and mortality following esophagectomy in ANZ to define optimal hospital caseload.

**Methods:**

As the standard of care following esophagectomy in ANZ is admission to an intensive care unit (ICU), the prospective ANZ Intensive Care Society Adult Patient Database was used to identify patients undergoing esophagectomy from 2005 to 2022. In‐hospital mortality was first determined for hospitals with annual caseloads defined as high (18+), medium–high (12–17), medium–low (6–11), and low (1–5). To define optimal caseload, mortality was also analyzed against hospital volume using piecewise linear regression and nonlinear (restricted cubic spline) methods.

**Results:**

Six thousand two hundred thirty‐four patients underwent esophagectomy in 161 hospitals. Twenty‐five percent of procedures were performed in low‐volume hospitals (*n* = 1558) and 19.9% in high‐volume hospitals (*n* = 1239). Overall, in‐hospital mortality ranged from 0.73% in the highest volume hospitals to 5.71% in the lowest volume hospitals. High‐volume hospitals also had a shorter length of stay in hospital (*p* < 0.001) and ICU (*p* < 0.001). The optimal annual hospital volume for the lowest mortality was identified as 21 cases per year. After adjusting for confounders in multivariable analysis, low‐volume hospitals showed the highest risk of mortality with ORs of 3.98 (low), 3.39 (medium–low), and 3.32 (medium–high) versus high‐volume (all *p* < 0.05).

**Conclusions:**

A positive volume–outcome relationship in ANZ was demonstrated for mortality following esophagectomy, with hospitals performing 21 or more surgeries per year delivering lowest mortality.

## Graphical abstract

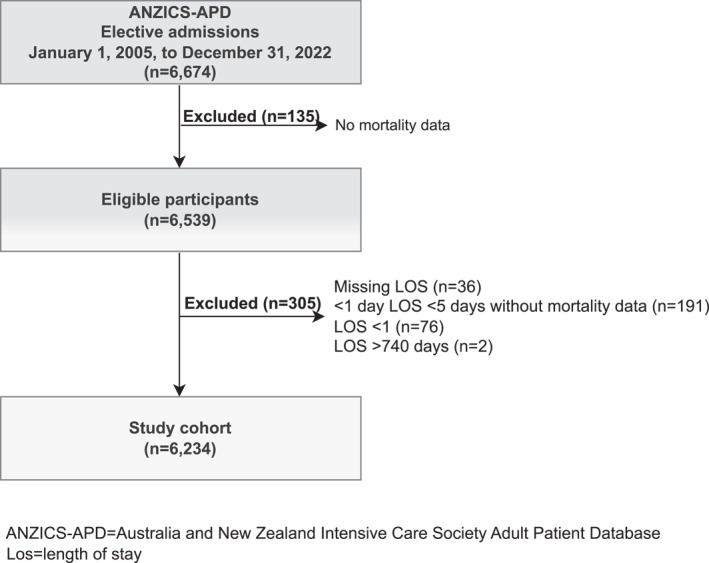

## 1 Introduction

Postoperative mortality is a marker of outcome quality following esophagectomy [1, 2, 3]. In 2024, the national esophago‐gastric cancer audit of England and Wales reported the 30‐day mortality rate was 1.4% and the 90‐day mortality was 3.0% for patients undergoing esophagectomy [4]. In an earlier Australian and New Zealand study, which analyzed hospital administrative data from 2008 to 2015, 30‐day and in‐hospital mortality rates of 2.1% and 3.1% for esophagectomy were reported [5]. This risk of postoperative death remains significant.

Postoperative mortality has declined over recent decades. A meta‐analysis postulated that this was related to multidisciplinary teams, more accurate preoperative staging and case selection, better perioperative management, and higher quality surgical care in high‐volume institutions [6, 7, 8, 9, 10]. Conversely, a 2011 study of 4498 patients from the United States (US) reported that a subset of low‐volume hospitals still achieved good outcomes [11]. This was attributed to higher nurse ratios and higher‐level facilities, which were not available in other small volume centers. Previous data from the United Kingdom (UK) and Europe supported centralization of services to high‐volume hospitals, although the definition of high‐volume differed in different countries [5, 9, 12, 13, 14, 15]. Furthermore, what might be appropriate in geographically smaller countries with high population densities might be difficult to deliver in geographically larger countries with smaller less dense populations such as Australia and New Zealand.

In the Netherlands in 2011, “high‐volume” was defined as more than 20 esophagectomies per year [9, 13]. This was redefined in 2021 to > 51 esophagectomies [8]. A 2015 study in the United Kingdom established the minimum volume for a “high‐volume” center as more than 26.4 esophagectomies per year [16]. Due to the relatively smaller populations in Australia and New Zealand, and the complexities of providing high‐level health services to populations dispersed over vast distances, volume definitions have been more conservative. Centralizing care to centers, which meet European high‐volume definitions, might not be acceptable as it would necessitate stopping esophageal cancer surgery in 3 of the 8 Australian States/Territories, and some patients would be required to travel more than 3,000 km to access care.

In Australian guidelines published in 2021, a high‐volume hospital was defined as performing 6 or more esophagectomies each year [17]. This was based on data from a previous study from Australia and New Zealand [5], a second study from the State of Queensland [12], and a third from New South Wales [18]. Access to a much larger clinical dataset from a binational clinical audit system with near complete coverage of the populations of Australia and New Zealand now provides an opportunity to reconsider the volume–outcome relationship and the definition of a high‐volume center for esophagectomy. This study analyzed these data to define the minimum annual surgical caseload for lowest postoperative mortality in Australia and New Zealand.

## 2 Materials and Methods

**FIGURE 1:**
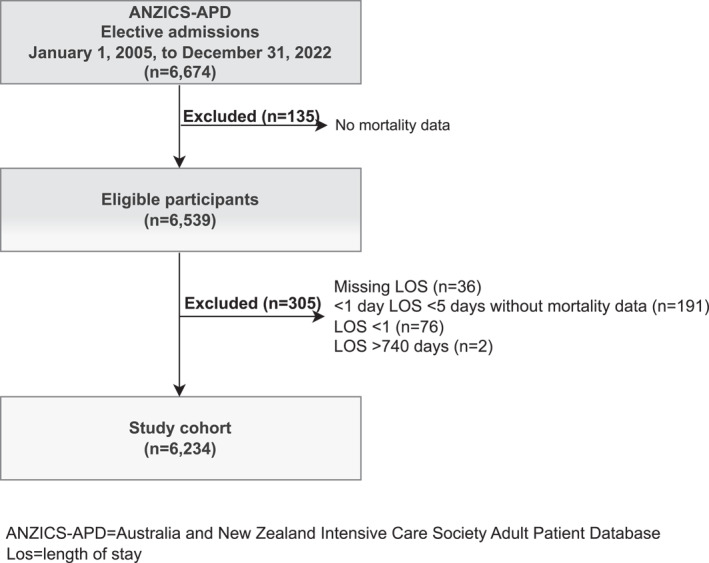
Flowchart of participant selection and inclusion in the study.

This study used prospectively collected data from the Australia and New Zealand Intensive Care Society (ANZICS) Adult Patient Database, a clinical quality registry of admissions to adult public and private intensive care units (ICUs) collected by the ANZICS Center for Outcome and Resource Evaluation [19]. This currently captures 99% of ICU admissions in Australia and more than 80% in New Zealand [20]. To ensure data completeness and accuracy, ANZICS database custodians have audited sites and apply validation rules to data collection from electronic medical records. As the standard of care for esophagectomy in Australia and New Zealand is admission to ICU following surgery, the ANZICS database captures almost all esophagectomies in Australia and New Zealand. De‐identified data for patients admitted electively following esophagectomy for cancer to ICU from January 1, 2005, to December 31, 2022 were obtained. Patients were excluded if they did not have a recorded length of stay (LOS), or where hospital LOS was 4 days or less unless death was recorded, or it was more than 740 days (Figure 1). The low‐trim point was applied as it was considered implausible patients who survived would stay for 4 days or less after esophagectomy. Patients who died at any time point, including within the first 4 days, were all included in the analysis.

The primary outcome was all‐cause in‐hospital mortality. Secondary outcomes included in‐ICU mortality and readmission to ICU within the same hospitalization, ICU and hospital LOS, and intubation. In‐hospital mortality was defined as death from any cause during the index hospitalization. Readmission to ICU within the same hospitalization was defined as those patients who had a return to ICU during that admission. In‐hospital LOS was defined as the total number of days spent in hospital. Similarly, ICU LOS was defined as the number of days spent in the ICU. Intubation was a binary outcome (yes/no) indicating whether re‐intubation was required after surgery.

Patient‐level covariates that might influence mortality were also included. These were as follows: sex, age, types of hospital admission (rural, metropolitan, tertiary, and private), comorbid conditions, and their Acute Physiology and Chronic Health Evaluation (APACHE) scores. “Tertiary” was defined as a public (government funded) hospital with cardiothoracic or neurosurgical services, a “metropolitan or rural/regional service” was a public hospital, which was not tertiary and based solely on geographic location, whereas “private” was defined as a private hospital irrespective of services offered or location. Comorbidities were defined as patients with concurrent diseases that included chronic respiratory, cardiovascular, liver, renal, endocrine, and immunological conditions. APACHE III scores, which combine three key factors—age scores, acute physiology scores, and chronic health scores, were used as a marker of seriousness of acute health events in ICU.

The exposure of interest was the hospital case volume, defined as the number of esophagectomies performed at each hospital each year. Each hospital each year was considered as a separate outcome variable. Hospitals each contributed a data point if they performed at least one esophagectomy in a year, but data were not contributed by a hospital, which did not perform an esophagectomy in that year. Hospitals were initially grouped according to the annual caseload, defined as high (18+), medium–high (12–17), medium–low (6–11), and low (1–5) volume. European definitions of high‐volume were not initially applied to define these groups. The categories were consistent with previous literature from Australia [5, 12, 18]. Twelve or more was defined as high‐volume in one previous study [5]. For the current study, this group was subdivided into 2 groups: a medium–high (12–17) group and a high (18+) group. As each hospital could vary in its volume across years, categories could change from year to year such that a medium–high‐volume hospital in 1 year would be considered a high‐volume in another year if operation numbers met the definitions applied below. Outcomes were compared across volume groups.

To determine the optimal number of esophagectomies for minimal mortality, volume was also evaluated as a continuous variable. Three analysis methods were employed: restricted cubic splines regression, kernel density estimation (KDE), and threshold‐based analysis using piecewise linear regression. Kernel density estimation, a nonparametric method, was applied to estimate the probability density function of mortality rates across different esophagectomy volumes [21]. A sequence of thresholds at 5‐unit intervals was also generated, and the corresponding mortality rate for each threshold was calculated. Following the threshold identification, a piecewise regression model was fitted to the data to identify significant changes in the slope of the mortality rate. Subsequently, to explore potential nonlinear relationships, a restricted cubic spline model was employed to determine the inflection point where the mortality rate started to decline significantly and then stabilized [22].

Statistical analyses involved univariate and multivariable logistic regressions to explore the association between hospital volume and in‐hospital and ICU mortality. We fitted models against the categories of volumes, adjusted for patient demographic and clinical characteristics (sex, age, and medical complexity for comorbidity), as well as hospital admission type (All covariates were included in each of the final multivariable models in addition to the primary variable of interest). Results of the multivariable logistic regressions were expressed as odds ratios (ORs), 95% confidence intervals (CIs), and *p*‐values.

Analyses were performed using SPSS Statistics (IBM SPSS Statistics for Macintosh, Version 28, Armonk, TY, USA) and R version 4.3.2 for Windows (R Core Team, R Foundation for Statistical Computing, Vienna, Austria). Hypothesis tests were two‐sided and *p*‐values < 0.05 were considered significant. Ethical approval for this study was obtained from the Alfred Human Research Ethics Committee. Project No: 432/23.

## 3 Results

**FIGURE 2:**
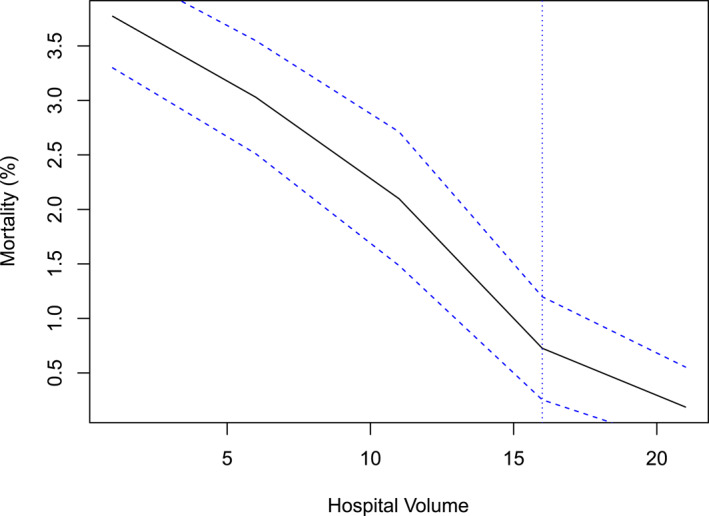
Threshold‐based hospital volume analysis with piecewise linear regression to determine the minimum annual mortality rate. The *Y*‐axis represents annual mortality rates for different volume thresholds, calculated by dividing the number of deaths by the total number of surgeries at each threshold. The blue dashed lines represent bounds of the 95% confidence interval. The dotted vertical line at *X* = 16 indicates the breakpoint where annual mortality is minimized.

From January 1, 2005, to December 31, 2022, data from 6674 patients admitted to ICUs following esophagectomy for esophageal cancer were collected. From these, 135 patients were excluded due to age less than 18 or no available mortality data and 305 were excluded with missing LOS data, LOS > 2 years, or < 5 days without a reported death. The final study cohort included 6234 patients. Esophagectomies were performed in 161 hospitals, and the in‐hospital mortality was 3.8% across the 17 years for the full cohort. There was a decline in in‐hospital mortality over time, from a peak in 2006 at 7.5% until 2015 after which mortality stabilized at around 3.4% (Supporting Information S1: Figure 1). The number of esophagectomies performed for each hospital volume category, the number of hospitals, and patient demographics are summarized in Table 1. Differences were seen between the different hospital volume cohorts for age, sex, and hospital type. There were also differences seen in comorbidities with high‐volume hospitals less likely to undertake esophagectomy in patients with significant cardiac and respiratory comorbidities but more likely to operate on individuals classified as having “endocrine and immunological” comorbidities—for example, diabetes (Supporting Information S1: Table 1). The geographic distribution of cases is summarized in Supporting Information S1: Figure 2.

**TABLE 1:** The number of esophagectomies by the hospital volume group caseload, hospital type, and patient demographics.

Factors	Total	High (18+)	Medium–high (12–17)	Medium–low (6–11)	Low (< 5)	*p*‐value
Number (%) of esophagectomies	6234 (100%)	1239 (19.9%)	859 (13.8%)	2578 (41.4%)	1558 (25.0%)	< 0.001
Number (%) of hospitals	161 (100%)	5 (3.1%)	5 (3.1%)	27 (16.8%)	124 (77.0%)	< 0.001
Sex (male)	4892 (78.5%)	1026 (82.8%)	671 (78.1%)	2026 (78.6%)	1169 (75%)	< 0.001
Age, mean (SD)	65.2 (10.4)	64.7 (9.7)	65.2 (10.8)	64.7 (10.1)	66.4 (11.0)	< 0.001
Hospital type						< 0.001
Metropolitan (*n* = 30)	945 (15.2%)	0	0	600 (23.3%)	345 (22.1%)	
Tertiary (*n* = 38)	3401 (54.6%)	855 (69.0%)	566 (65.9%)	1510 (58.6%)	470 (30.2%)	
Private (*n* = 60)	1643 (26.4%)	384 (31.0%)	293 (34.1%)	468 (18.2%)	498 (32.0%)	
Rural/Regional (*n* = 33)	245 (3.9%)	0	0	0	245 (15.7%)	

Abbreviation: SD = standard deviation.

Outcomes for the different volume categories are summarized in Table 2. High‐volume hospitals had the lowest mortality and shorter ICU and hospital LOS. Postoperative mortality in high‐volume hospitals was 0.73% compared to 4.07% in medium–high, 3.96% in medium–low, and 5.71% in low–volume hospitals. High‐volume hospitals were less likely to ventilate a patient after surgery or readmit a patient after discharge from ICU.

**TABLE 2:** Univariate analysis of surgical outcomes in different hospital volume groups.

Outcomes	Overall	High (18+)	Medium–high (12–17)	Medium–low (6–11)	Low (< 5)	*p*‐value
Primary: Number of deaths during admission (number [rate])	235 (3.8%)	9 (0.7%)	35 (4.1%)	102 (4.0%)	89 (5.7%)	< 0.001
Secondary: Number of deaths during ICU admission (number [rate])	100 (1.6%)	2 (0.03%)	14 (0.2%)	37 (0.6%)	47 (0.8%)	< 0.001
Hospital length of stay (days)^a^	20.29 (14.00:19.80–20.80)	18.60 (13:17.10–20.11)	21.51 (15:20.20–22.80)	20.51 (14:19.80–21.30)	20.60 (14:19.51–21.70)	0.014
ICU length of stay (days)^a^	4.22 (2.77:4.07–4.37)	3.33 (2.10:3.09–3.58)	3.59 (2.10:3.29–3.89)	4.40 (2.90:4.15–4.64)	4.99 (2.90:4.63–5.34)	< 0.001
Intubation (number [rate])	1519 (24.4%)	249 (20.1%)	252 (29.3%)	578 (22.4%)	440 (28.4%)	< 0.001
Readmission (number [rate])	100 (1.6%)	2 (0.03%)	14 (0.2%)	37 (0.6%)	47 (0.8%)	0.008

Abbreviation: ICU = intensive care unit.

^a^ Mean (median: 95% CI).

**FIGURE 3:**
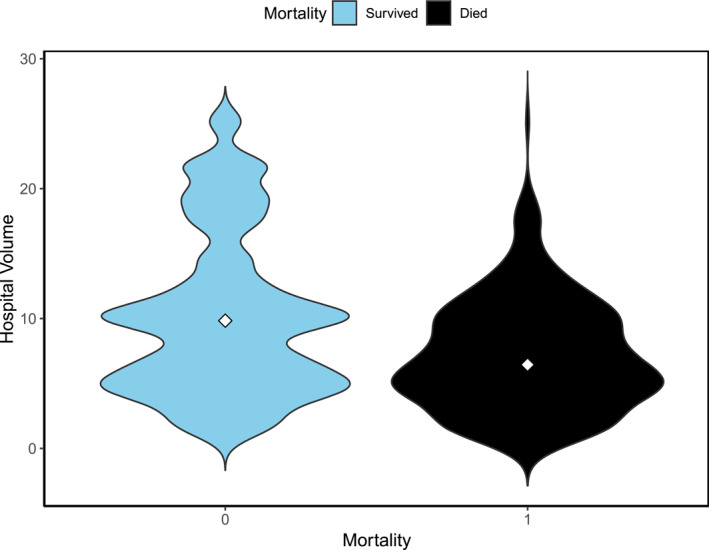
Violin plot, which combines kernel density estimation and the box plot, offers a detailed view of the distribution of surgery volumes for both outcomes with the median score in the median marked in white in the middle. The plot suggests that the optimal surgery volume threshold for minimizing mortality is 21 operations per year.

**FIGURE 4:**
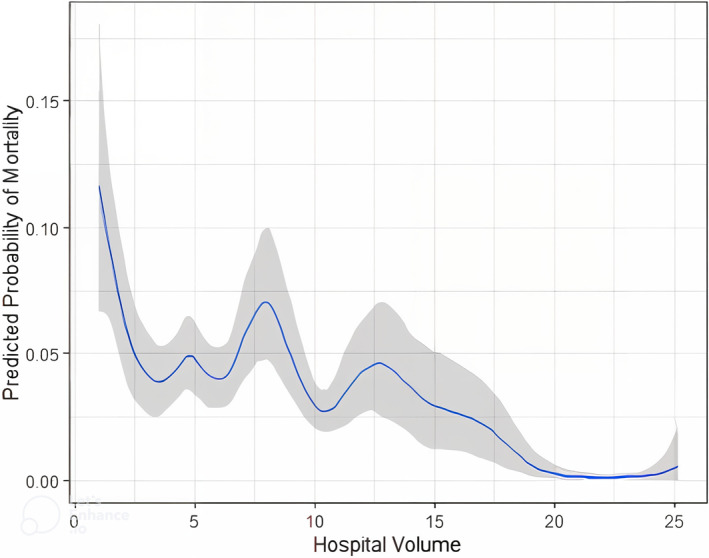
Hospital volume versus predicted probability of mortality. This plot illustrates the relationship between hospital volume and the predicted probability of mortality, modeled using restricted cubic splines. The analysis identifies the inflection point where mortality rates begin to decline significantly as hospital volume increases. The shaded area represents the 95% confidence intervals for the predicted probabilities.

The annual mortality for different surgery volume thresholds was calculated to identify the threshold where the annual mortality was minimized (Figures 2 and 3). The piecewise linear regression and KDE initially determined that 16 esophagectomies per year was the threshold for lowest annual mortality (Figure 2). However, when a restricted cubic spline model, which made no assumptions about parametric data distribution, was applied, 21 esophagectomies per year were determined to be the optimal volume threshold for achieving lowest postoperative mortality (Figure 4).

The median hospital LOS for patients who survived to discharge was 14 days and the mean LOS was 20.3 days (Table 2). High‐volume hospitals had the shortest LOS (mean 18.6 days). The median ICU LOS was 7 days and the mean ICU LOS 10.8 days (range 5–89). High‐volume hospitals also had the shortest ICU LOS (mean 10.3 days).

Multivariable logistic regression analysis showed that smaller hospital volumes were associated with higher in‐hospital mortality. Compared to high‐volume hospitals, low‐volume hospitals exhibited an odds ratio (OR) of 3.98 (95% CI: 1.60–10.09 and *p* = 0.004), medium–low an OR of 3.39 (1.39–9.12 and 0.010), and medium–high an OR of 3.32 (1.22–9.76 and 0.022) for postoperative mortality (Supporting Information S1: Figures 3 and 4).

Other factors shown to be associated with increased in‐hospital mortality included comorbidities (OR of 1.88 (1.29–2.71 and < 0.001)) and a higher APACHE III score (OR = 1.05, 1.04–1.06, and < 0.001). Other variables, including age (OR = 1.01, 0.99–1.03, and 0.50), sex (OR = 1.21, 0.79–1.89, and 0.40), and hospital classification (e.g., private hospitals OR = 0.65, 0.35–1.22, and 0.20) were not associated with in‐hospital mortality.

## 4 Discussion

This study confirmed a relationship between hospital volume and mortality for esophagectomy in Australia and New Zealand, with hospitals performing 21 or more esophagectomies for cancer each year delivering the lowest mortality and shorter hospital and ICU stays. This is consistent with recommendations from the Netherlands, Germany, and the United Kingdom [8, 15, 23]. The mortality rate for hospitals performing at least 18 esophagectomies per year was lowest at 0.73% compared to 4.07% for medium–high, 3.96% for medium–low, and 5.71% for low‐volume hospitals. This mortality rate in high‐volume hospitals is less than reported contemporary mortality rates from the United Kingdom [4] and Netherlands [24], countries where all hospitals exceed our high‐volume threshold. Reduced lengths of stay and complications were also delivered by high‐volume hospitals in our current study.

A previous analysis of United Kingdom data from 2005 and 2010, which applied a “high‐volume” definition of at least 26.4 esophagectomies per year, concluded that case volume was an independent predictor of mortality [16]. That study also reported an in‐hospital mortality rate of 4.2%, which was similar to the in‐hospital mortality of 3.8% seen across our entire cohort. The UK healthcare system has restricted the number of centers performing curative esophageal surgery since 2001 and seen a doubling of the average annual surgical volume, and significant decreases in 30‐day, 90‐day, and 1‐year mortality and the length of stay [25].

The Netherlands started a similar centralization process in 2000 [26]. Analysis of data from the Dutch Upper Gastrointestinal Cancer Audit in 2016–2019 defined a threshold minimum of 20 esophagectomies per center per year and also found that outcomes continued to improve until a plateau of 50–60 operations per year was reached [8]. In the Netherlands, 51 cases per year is now considered to be the minimum volume for centers performing esophagectomy [8]. In Germany, an analysis of 22,700 complex esophageal surgeries over 5 years from 2010 to 2015 recommended a minimum volume threshold of 26 cases, and when applied, in‐hospital mortality decreased from 12.2% to 6.8% [15]. The difference in mortality outcome in that study was attributed to the improved management of complications in a high‐volume center and a “failure to rescue” in lower volume centers. However, it should be noted that the in‐hospital mortality rate of 6.8% in high‐volume German hospitals was nearly double the 3.8% rate for the entire cohort in our study and nearly 10‐fold higher than the 0.73% in the high‐volume hospitals in our study.

Guidelines published in 2021 defined an Australian high‐volume hospital as performing six or more esophagectomies per year [17]. This recommendation was based on administrative data from two Australian States [12, 18] and a data source which collected data from less than 50% of hospitals in Australia and New Zealand [5]. The study of 908 patients who underwent surgery from 2001 to 2008 in New South Wales reported the 30‐day mortality was 4.1%, with the 5‐year absolute survival better for patients undergoing surgery in centers undertaking 6 or more esophagectomies per year [18]. A subsequent study of 1167 patients from 2001 to 2015 in Queensland identified 30‐day mortality following esophagectomy ranged from 0.0% to 2.4%, with lower rates seen in higher volume centers [12]. This study also defined high‐volume as more than 6 procedures per year. The third study included 2252 patients from 2008 to 2015 across Australia and New Zealand and found an overall in‐hospital mortality rate of 3.1%, and again the highest volume hospitals had the lowest mortality, with high‐volume defined as 12 or more operations [5]. As our current study collected clinical data from a much larger cohort of 6234 patients, we were able to more accurately determine optimal outcomes that were delivered by hospitals performing at least 21 operations per year.

However, there is likely a problem with our conclusion that optimal outcomes will be achieved by ensuring esophagectomies are only performed in hospitals in Australia and New Zealand that perform at least 21 cases per year. Achieving this would require restructuring the delivery of esophageal cancer services across most of Australia and New Zealand, with the consolidation of services to fewer centers in large capital cities. This would require the cessation of services in many smaller population regions, including the State of Tasmania and the Northern Territory, and some patients would then need to travel much larger distances for surgery. It is likely that a minimum population of 700,000 to 1,000,000 people would be needed to support each high‐volume center, and this would require consolidating from 161 hospitals identified in the current study to no more than 40 hospitals.

A major challenge for this in Australia and New Zealand is low population density. Australia has a land mass similar in size to USA and larger than Europe, but with more than 60% of its population of 27.7 million people living in only 5 cities. Hence, many potential patients are remotely located and need to travel significant distances to reach these cities. A real example is that most individuals requiring an esophagectomy for cancer who currently live in Darwin at the top of Australia currently travel more than 3000 km to the city of Adelaide in South Australia.

Our study has some limitations. Data capture might not have been complete, and not all variables were captured across all the years as not all hospitals recorded information through ANZICS‐APD across all 17 years of our study period. This means that some patients undergoing surgery in some hospitals, particularly in earlier years, were missed. However, the database now captures > 99% of admissions to ICUs across Australia and more than 80% in New Zealand with only a small number of patients missed in more recent years. Additionally, as the database had an ICU outcome focus, it was limited in some of the information stored. Consequently, we did not have access to details about the tumor characteristics, the type of surgery, and if any neoadjuvant treatments had been used. Further, any patient who died in the operating room following a catastrophic event during the actual operation would not have reached ICU and therefore would have been missed by our study. However, as death on the operating table during esophagectomy is exceptionally rare, this limitation is unlikely to have impacted the minimum caseload determined in the current study.

We also did not have the ability to cross‐reference the cohort in this study against any other national registry or audit that could also be used to confirm the number of esophagectomies performed. A national registry that provides these type of data does not exist in Australia and New Zealand. However, we were able to identify from cancer registries the number of new esophageal cancer diagnoses in Australia and compare that with the volume of surgical patients identified using the ANZICS database. As we have previous shown that approximately 30% of patients diagnosed with esophageal cancer undergo esophagectomy, we were reassured that the number of cases identfied each year in the current study are consistent with the number of new cancer diagnoses [27].

Our analysis was also limited to hospital volume but not surgeon volume. Individual surgeons could not be identified from the database. Additionally, in Australia, it is not unusual for “high‐volume” surgeons in some regions to work across multiple hospital sites in the public and private sectors, and as a result, an individual surgeon's work can be spread across several lower volume centers. This nuance is not captured in our analysis, and conclusions are limited to individual hospitals and the systems they implement. For now, we suggest that it is not just the surgeon, but rather the whole team that counts when aiming for best outcomes after esophagectomy.

Despite these limitations, our findings on the impact of hospital volume on mortality and length of stay are likely to be robust and they support centralization to sites which undertake 21 or more esophagectomies each year. This is consistent with analyses from European countries. A strength of our study is that the data source used was a clinical database, which was designed to collect clinical outcomes and provide data to clinicians. The data obtained and analyzed represents the largest and most comprehensive dataset for esophagectomy available for Australia and New Zealand. Other studies evaluating volume versus outcome have used administrative data [5, 12, 18].

Consistent with international studies, a positive volume–outcome relationship was demonstrated for mortality following esophagectomy for cancer, with higher volume hospitals delivering lowest mortality across Australia and New Zealand. The minimum number of esophagectomies required for the lowest mortality rates in this setting was 21. The challenge to achieving this will be to redesign healthcare systems to centralize esophageal cancer surgical care and to address the impact of geography on delivering safe outcomes to smaller populations dispersed across large countries.

## Author Contributions

**Josipa Petric:** conceptualization, data curation, formal analysis, funding acquisition, investigation, methodology, project administration, resources, software, visualization, writing – original draft, writing – review and editing. **Muktar Ahmed:** data curation, formal analysis, methodology, software, validation, writing – review and editing. **Maziar Navidi:** conceptualization, formal analysis, investigation, methodology, supervision, writing – original draft. **David Pilcher:** conceptualization, data curation, formal analysis, investigation, project administration, software, supervision, validation, visualization, writing – original draft, writing – review and editing. **Shailesh Bihari:** data curation, formal analysis, investigation, project administration, supervision, writing – original draft, writing – review and editing. **Norma B. Bulamu:** conceptualization, data curation, methodology, supervision, writing – original draft. **Tim Bright:** conceptualization, investigation, methodology, project administration, supervision, writing – original draft, writing – review and editing. **David I. Watson:** conceptualization, formal analysis, investigation, methodology, project administration, supervision, visualization, writing – original draft, writing – review and editing.

## Ethics Statement

Ethical approval for this study was obtained from the Alfred Human Research Ethics Committee. Project No: 432/23.

## Conflicts of Interest

The authors declare no conflicts of interest.

## Supporting information

Supporting Information S1
