# Supplementary material for: Optimal Hospital Volume to Minimize Postoperative Mortality After Esophagectomy for Cancer in Low Population Density Countries: A Binational Study of Australia and New Zealand
Source: World J Surg. 2025 Apr 17;49(6):1537–45. doi: 10.1002/wjs.12595 (PMC12134189; doi:10.1002/wjs.12595)
Supplement: Supplementary file 1 — Supporting Information S1 [file WJS-49-1537-s001.docx]

**SUPPLEMENTARY FILE**

**Title:** Optimal hospital volume to minimise postoperative mortality after esophagectomy for cancer in low population density countries: a binational study of Australia and New Zealand.

**Authors:**

Josipa Petric,^1^ MD, BMedSc, MS

Muktar Ahmed,^1^ BSc, MPH, PhD

Maziar Navidi,^2^ MBChB, MD, FRCSEd

David Pilcher,^3-5^ MBBS MRCP(UK) FCICM FRACP

Shailesh Bihari,^1,6^ MBBS, MD, FCICM, PhD

Norma B Bulamu,^1^ BPharm, MPH, PhD

Tim Bright,^1,2^ MBBS, MS, FRACS

David I Watson^1,2^ MBBS, MD, PhD, FRACS, FRCSEd (Hon), FAHMS

^1^Flinders Health and Medical Research Institute and College of Medicine and Public Health, Flinders University, South Australia, Australia.

^2^Department of Surgery, Flinders Medical Centre, South Australia, Australia.

^3^Department of Intensive Care, Alfred Health, Victoria, Australia.

^4^The ANZICS Centre for Outcome and Resources Evaluation, Victoria, Australia.

^5^The Australian and New Zealand Intensive Care Research Centre, School of Public Health and Preventive Medicine, Monash University, Victoria, Australia.

^6^Intensive and Critical Care Unit, Flinders Medical Centre, South Australia, Australia.

***Correspondence to:**

Professor David I Watson

Head, Flinders University Department of Surgery

Room 3D211, Flinders Medical Centre

Bedford Park

South Australia 5042

AUSTRALIA

Ph: +61 8 8204 6086

Fax: +61 8 8204 6130

Email: david.watson@flinders.edu.au

**Supplementary table 1.** Association of Comorbidities with Hospital Volume Categories

| **Comorbidities** | **Total** | **High (18+)** | **Medium-high (12-17)** | **Medium-low (6-11)** | **Low (<5)** | ***P-value** |
| --- | --- | --- | --- | --- | --- | --- |
| Cardiovascular | 255 | 14 (1.1%) | 40 (4.7%) | 116 (4.5%) | 85 (5.5%) | <0.001 |
| Respiratory | 197 | 19 (1.5%) | 28 (3.3%) | 94 (3.6%) | 56 (3.6%) | <0.001 |
| Liver and renal | 25 | 9 (0.7%) | 8 (0.9%) | 31 (1.2%) | 21 (1.3%) | <0.001 |
| Endocrine and immunological | 752 | 178 (14.4%) | 80 (9.3%) | 309 (12.0%) | 185 (11.9%) | <0.001 |

*P-values are derived from the Chi-square test.


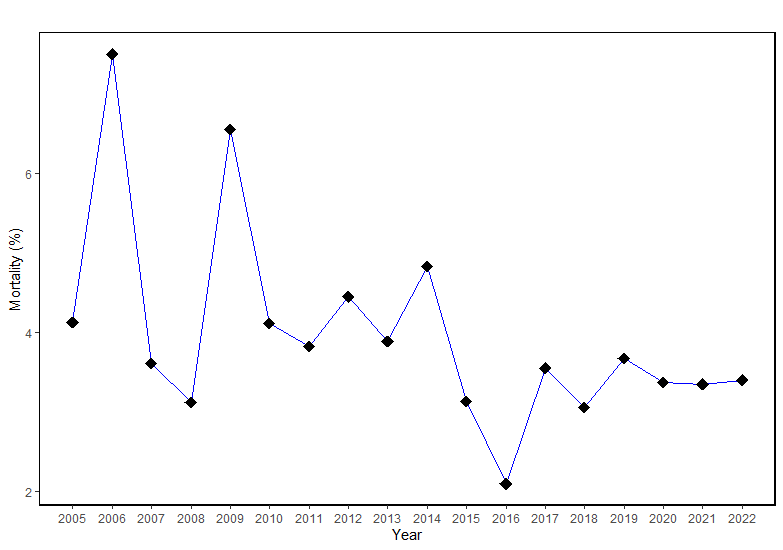


**Supplementary Figure 1.** Yearly in-hospital mortality across all hospitals in Australia and New Zealand from 2005 to 2022.


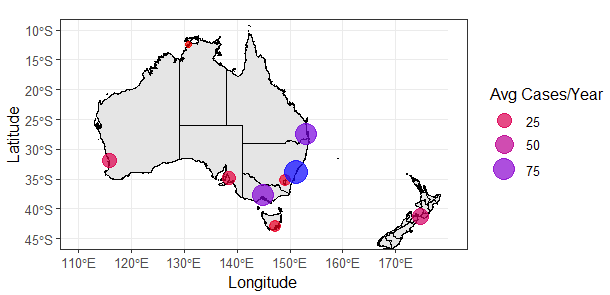


**Supplementary Figure 2.** Geographical distribution of average annual caseloads in Australia and New Zealand. The size of each dot represents the average annual caseload at each centre, with larger dots indicating higher caseload volumes.

**
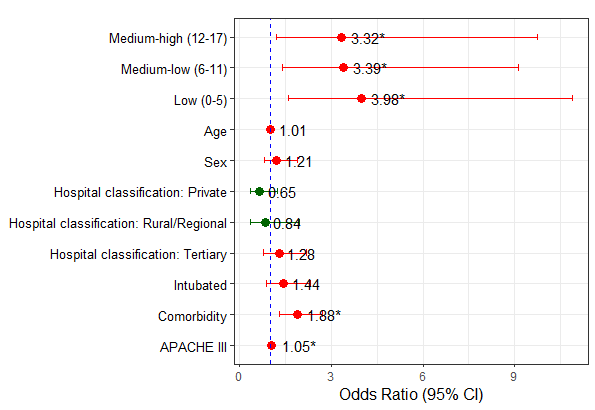
**

**Supplementary Figure 3.** The forest plot illustrates the odds ratios (OR) with 95% confidence intervals (CI) for various characteristics associated with in-hospital mortality. Characteristics with ORs significantly associated with in-hospital mortality (p < 0.05) are marked with an asterisk (*).

**
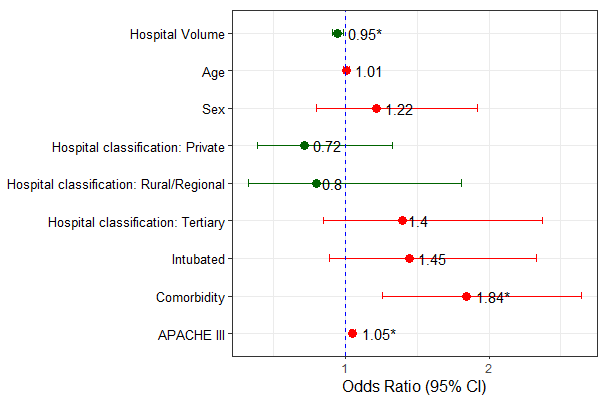
**

**Supplementary Figure 4.** Adjusted estimates for hospital volume and mortality, accounting for various covariates. Hospital volume is considered as a continuous variable in this analysis. Characteristics with ORs significantly associated with in-hospital mortality (p < 0.05) are marked with an asterisk (*).
